# A Qualitative Evaluation of Social Aspects of Sugar-Rich Food and Drink Intake and Parental Strategies for Reductions

**DOI:** 10.3390/ijerph191811647

**Published:** 2022-09-15

**Authors:** Bodil Just Christensen, Sidse Marie Sidenius Bestle, Ellen Trolle, Anja Pia Biltoft-Jensen, Jeppe Matthiessen, Anne Dahl Lassen

**Affiliations:** National Food Institute, Technical University of Denmark, Kemitorvet Building 201, 2800 Kongens Lyngby, Denmark

**Keywords:** family-based intervention, parenting, behavioural reduction strategies, dietary guidelines, Denmark, pre-school children, qualitative interviews

## Abstract

Danish children have a much larger intake of sugar-rich foods and drinks than recommended. This study aimed to (1) explore social aspects and practices of pre-school children’s intake of sugar-rich foods and drinks and (2) evaluate barriers and parental strategies to reduce their children’s intake of sugar-rich foods and drinks employed in connection with the 3.5-month family-centred intervention trial ‘Are you too sweet?’. Intervention components included communication of the recommended maximum intake and reduction strategies, supported by resources encouraging and facilitating behavioural changes. A random sample of families (n = 24) from intervention schools participated in post-intervention semi-structured interviews. A thematic content analysis was conducted, revealing three main domains of social practices: (1) ‘family treats’, including the weekly Danish concept ‘Friday sweets’, (2) ‘everyday treats’, such as sweet snacks in lunch packs, between-meals snacks and soft drink habits and (3) ‘socialized treats’, including treats at special occasions. Parents employed several strategies, most often substitution and portion-size reduction, but also limiting home availability. Families most frequently made changes that were easily adoptable and close to existing routines at home. In conclusion, the intervention components provided families with knowledge and strategies that facilitated behavioural changes towards reducing the intake of sugar-rich foods and drinks.

## 1. Introduction

Within public health research and policies, there is widespread consensus that reducing the intake of sugar-rich foods and drinks holds important potential for preventing obesity and related diseases [1,2,3], poor diet quality [4,5] and dental caries [6]. Interventions and policies frequently target children and adolescents in order to influence and shape preferences and eating patterns that may persist into adulthood [7,8]. Parents have a central role in the development of their children’s food habits. As gatekeepers for providing food and feeding practices, parents, to a large extent, determine a child’s eating behaviour [9]. Furthermore, eating patterns are interconnected with social meanings and norms, food values, identity markers and perceptions of health and well-being [10,11,12]. Hence, the literature shows that interventions that target behavioural changes in relation to diet may well benefit from including social aspects that could facilitate or impede such changes [13,14]. 

According to data from the Danish national survey of diet and physical activity, Danish children’s intake of sugar-rich products largely exceeds recommendations [15], and sales data suggest that on average every Dane obtains 20% of their daily energy from sugar-rich foods and drinks [16]. Previous studies have documented a lack of knowledge on maximum intake limits for sugar-rich foods and drinks among parents [17,18]. Consequently, new guidelines on the maximum intake of sugar-rich foods and drinks were developed to diminish or even close this knowledge gap [16], along with a range of information materials [19].

Despite numerous studies of parental attitudes, approaches and practices, it remains unclear which strategies are the most effective at securing a diet low in sugar-rich foods and drinks [20,21]. Structure-based parenting strategies aimed at structuring the home environment (setting clear rules, implementing fixed routines, offering supportive guidance, etc.) have been suggested as effective in securing a healthy eating pattern [22,23] and in relation to reducing intake of sugar-rich foods [24]. Building on these results, Savage and colleagues tested eight different combinations of parental strategies to moderate children’s (6–9 years of age) intake of sweets and found that parent-shared decision-making, including shared decision-making between parent and child on routines concerning sweets, appeared to be the most helpful component in moderating children’s intake [25]. Thus, some effective strategies have been identified, but several authors state that further research is needed to determine effective strategies [20,21,24,25].

In order to increase awareness and reduce the high intake of sugar-rich foods and drinks, the ‘Are you too sweet?’ intervention targeting 5–7-year-old children and their families was developed (See Bestle et al. 2020 [26]). A family-focused approach where the home environment was the key target was chosen both due to the above arguments that parental involvement has been found to be an important contributor to successful interventions [27] and due to surveys documenting high intakes during weekends especially [15]. The primary outcome, reductions in the intake of sugar-rich foods and drinks, will be reported separately. The aims of the present sub-study are to (1) explore the social aspects and practices of sugar-rich foods and drinks (‘sweet treats’) consumed by pre-school children; and (2) evaluate barriers and parental strategies employed to reduce children’s intake of sugar-rich foods and drinks. 

## 2. Materials and Methods

### 2.1. Setting and Intervention Design

This qualitative study was a part of the intervention ‘Are you too sweet?’. The design and methods of the intervention have been described in Bestle et al. 2020 [26]. Briefly described, the setting for the intervention was the suburban municipality of Hvidovre, located near Copenhagen, Denmark. It was designed as a 3.5-month, cluster-randomized control trial, where 153 families with pre-school children aged 5 to 7 years were enrolled. The children and their families were randomized to either the intervention group (four schools, n = 95) or control group (two schools, n = 53). The aim of the intervention was to communicate new guidelines on the intake of sugar-rich foods and drinks, increase parental self-efficacy and behavioural capacity and thereby facilitate a reduction in children’s intake of sugar-rich products. Intervention messages were supported by a range of knowledge-building components and proposed strategies for behavioural change, and they were delivered at a school health nurse consultation. For a comprehensive description of the design of the main intervention, its theoretical background and the intervention components, see Bestle et al. 2020 [26], and Christensen et al. 2022 [19].

### 2.2. Intervention Components: School Health Nurse Consultation and Home-Use Materials

New official guidelines on the maximum intake of sugar-rich foods, drinks and salty snacks have recently been developed [16,28]. The aim of the guidelines was to provide easy-to-grasp and intuitively functional rules of thumb for adults and children. Hence, the guidelines were defined as a number of recommended maximum weekly servings of sugar-rich foods and drinks for all age groups. For children aged 4 to 6 years, the guidelines advise a maximum of four weekly servings of sugar-rich foods and drinks (for details, see Christensen et al. 2022 [19]). 

The intervention evolved around the school health nurse consultation that is mandatory to offer in Danish pre-schools. In the intervention group, the consultation was extended by 5 min targeting the families’ sugar intake habits. Through a dialogue-based approach, the school health nurse informed the family about maximum limits and possible changes with a focus on the child’s individual habits. The school health nurse’s advice and guidance were based on a preceding registration of the child’s intake of sugar-rich foods and drinks, enabling individualized guidance [29]. 

At the consultation, each family received a box with home-use tools comprising a booklet with visualizations of the maximum limits, suggestions for behavioural change strategies and tips and tricks for reductions, a deck of cards that could be used for different game activities, a read-aloud children’s book on dental health, a serving size board with reusable stickers illustrating the maximum recommended amount of servings, suggestions for local family activities, three small posters and stickers with the project logo. The aim of the home-use tools was to convey information and strategies in a positive, non-offensive tone of voice to facilitate intake reductions and support behavioural change in the family. As complementary components, an educational app with learning games on dietary and dental health was developed and provided to the participating families to supplement and support the tools in the home-use box. Furthermore, the families were invited to subscribe to the private Facebook^TM^ group ‘Sweet friends of Hvidovre’, used to provide parents with information and ‘reminders’ and build peer-to-peer support among participants. Thus, a broad range of supportive resources was made available to families in a flexible framework where they could select tools, tips and tricks according to their individual needs. 

### 2.3. Qualitative Interviews: Recruitment, Data Material and Analysis

The recruitment of interviewees was conducted with the sample size goal of approximately 25 participants to secure data saturation [30] and in order to cover a broad range of perspectives and family habits. A total of 35 families were contacted through random sampling taking into account school affiliation to obtain a balanced distribution among the four intervention schools; 11 contacted families declined the interview, mainly due to lack of time. Initially, 25 interviews were scheduled; we were able to conduct full interviews with 24 of these families. When possible, the participating child’s father or both parents were encouraged to participate in the interview in order to obtain a sample including both mothers and fathers. This was done because even if mothers often remain responsible for food parenting [31], fathers are increasingly involved in childcare and household chores [32], notably in a Danish context [33]. 

The primary author conducted all interviews using Microsoft Teams (video conferencing software, version 1.5.00.21668) (Microsoft. Redmond, Washington, DC, USA), as COVID-19 restrictions inhibited interviews in participants’ homes. Interviews were semi-structured [34], employing an interview guide with open-ended questions (see Supplementary Materials). Topics included knowledge about and adoption of new guidelines on sugar-rich foods and drinks in the family and in relation to the child, use and assessment of the intervention components (the box with home-use tools, the educational app and the Facebook group) and the family’s perceptions of family time and values. Furthermore, practices and changes regarding food and notably sugar-rich foods and drinks during the intervention period were assessed. Interviewees were encouraged to speak freely and include issues that were of importance to them. All interviews were recorded and transcribed verbatim. 

A thematic content analysis was employed [35]. The analysis was conducted according to the following steps: (1) The researcher who had conducted all of the interviews made a preliminary coding framework consisting of themes relevant to the project evaluation, i.e., perception of the guidelines on the maximum intake of sugar-rich foods and drinks, perceived reductions in different sweet treat categories, evaluation of the intervention components, barriers to behavioural change and how the COVID-19 pandemic had impacted family life (see Supplementary Materials for details). (2) The researcher who had conducted all of the interviews and another researcher independently analysed five randomly selected transcripts on the basis of the preliminary coding framework and refined the framework through two rounds of independent coding. (3) To establish coding reliability, an intercoder reliability test was performed in line with the measures proposed by Campbell [36]. Two reliability tests were conducted, as the first test yielded 77% agreement and led to further elaboration of the coding scheme, while the second reliability test provided 86% agreement and was evaluated as satisfactory. (4) Coding of all transcripts was conducted by one researcher using the NVivo software (Version 10, QSR International, Montreal, QC, Canada). 

## 3. Results

### 3.1. Interviews and Participant Characteristics 

Interviews lasted between 44 and 77 min, with an average length of 61 min. Nineteen single and five double (both parents) interviews were conducted (Table 1). Interviewees were more commonly mothers (n = 23) than fathers (n = 6), and interviewees’ educational level varied between basic school (13%), vocational education (29%) and short, medium or long higher education (17%, 21% and 21% respectively). There was an even distribution of parents to boys and girls among the interviewees, and a diverse range of family types was recruited (for further details, see Christensen et al. 2022 [19]).

### 3.2. Social Aspects of and Practices around Sweet Treats

In the analysis, four key themes and a range of sub-themes were identified (Table 2).

Furthermore, a pattern of social practices around sugar-rich foods and drinks emerged that included three different framings of practices related to (1) ‘family treats’, (2) ‘everyday treats’ and (3) ‘socialized treats’. ‘Family treats’ are sugar-rich products consumed in the household with close relatives to create family time, while ‘everyday treats’ comprised sweet snacks and soft drinks linked to everyday life, e.g., afternoon snacks or in the child’s lunch packs, and ‘socialized treats’ included sugar-rich servings at social occasions and celebrations, e.g., birthdays with family or classmates and festive and seasonal gatherings. Different strategies and barriers pertained to these three social contexts and related to the amount, frequency, type and function of the serving of sweet treats. In the following sections, practices, strategies and barriers will be described.

#### 3.2.1. ‘Family Treats’: Creating Family Time with Sugar-Rich Foods and Drinks

In Denmark, especially Friday night, but also Saturday and Sunday are culturally codified as ‘family time’. On Fridays, many families go to grocery stores or sweet shops and buy pick ‘n’ mix sweets for ‘fredagsslik’, which means ‘Friday sweets’. Treats are bought for both children and adults, and the valued tradition was spelled out in the interviews.

*“Friday sweets are simply a tradition. It has just always been that way, so that would be hard to change. Whether they get an ice cream on a Tuesday or a Wednesday or not at all, that, I think, would not be very important to them. But the Friday sweets are really—it is kind of sacred”*,

Mother to boy at school A.

Thus, many parents cherished the family ‘ritual’ of Friday sweets; they looked forward to it, and so did the children.

*“You look forward to all those things, or at least [name of the child] does, to arrange it nicely and have a good and cosy time with it”*,

mother to boy at school D.

One of the reasons why Friday sweets became valued among both adults and children was that on Fridays they are ‘allowed’, in the sense that a common strategy is to limit one’s intake of sweets and sugar-rich foods and drinks, and thus reserve it for weekends and holidays.

*“We have simply declared: “now it is limited to Friday sweets, and that is it”, and he seems to take it quite well”*,

mother to boy at school A.

Some families had chosen not to follow the tradition of Friday sweets as a culturally codified, fixed weekly tradition, but ‘family time’ was still closely related to sweet treats.

#### 3.2.2. Strategies for Behavioural Changes in Relation to ‘Family Treats’ 

Strategies for reductions related to Friday sweets and other sweet treats served to create family time were often dependent on existing family rules, e.g., whether families already had decided to omit sugar-rich foods and drinks during the week.

*“We have rules that at least on weekdays there are no sweets”*,

father to girl at school C.

The Friday sweets practice as such was not questioned in the large majority of families, but the amount and serving sizes were evaluated and at times reduced.

*“I am glad we have cut back on the intake on Fridays compared to earlier, where it was a huge pile of sweets and then a stomach ache the next day”*,

mother to girl at school D.

Encouraged by intervention materials, some of the families had changed their serving modus, e.g., to portion out servings in small bowls or cups to be aware of and limit how much the children were consuming; this pertained to both Friday sweets and other sweet treats served to create family time. One family changed their Friday habits altogether by introducing ‘Friday fruits’.

*“Now we have Friday fruits. The kids chip in what fruits we should buy and then they have something a little extra, like cream biscuits or a pancake or something like that, but we always have fruits and something sweet, but it is never sweets”*,

mother to girl at school C.

However, the most common strategies to limit ‘family treats’ were to reduce the portion sizes or to replace (some of the) chocolate, toffee or sweets with healthier alternatives, such as fruits. 

#### 3.2.3. ‘Everyday Treats’: Sugar-Rich Snacks and Soft Drinks Linked to the Everyday

All of the children in the study brought their own lunch packs for lunch in school. Children thus ate what their parents prepared at home. Lunch packs typically consisted of open-faced sandwiches (rye, wholegrain or white bread), vegetables and fruits, and parents often added snacks (salty or sweet) as small treats. At two intervention schools, there were rules for foodstuffs not allowed in lunch packs, e.g., cake, sweets or chocolate bars and cordial; the two other schools had no such restrictions.

‘Everyday treats’ also included sugar-rich snacks given in the afternoon. In many families, it was common for the child to have a light meal or a snack in the afternoon at home to keep them going until dinner or, e.g., on the way to an after-school activity. This is consistent with snacking patterns in other countries [37] and is comparable to British afternoon tea or Le Goûter in France, but unlike those often highly culturally codified servings, the content of the light afternoon meals in Denmark is not culturally predefined. What was served differed greatly between families.

*“I used to prepare a snack platter for him in the afternoon. I guess it was more sweet things than fruit and vegetables. It could be a small bowl with crisps, some chocolate or a chocolate bar. Or it could be a fruit stick”*,

mother to boy at school B.

A third practice related to ‘everyday treats’ was the consumption of soft drinks. Among interviewees, there was clear pattern as to whether families were drinking soft drinks or not. Only a subsample served carbonated soft drinks for their children, and as a general rule, these parents drank carbonated soft drinks themselves on a daily basis. In a few families, children had cordial, while the parents had soda, reflecting a view of cordial being mainly ‘for kids’.

*“We drink a lot of milk, they have water at school and in kindergarten, and then they can have one glass of cordial for dinner. Well, that is how we do it in our family, right?”*,

mother to girl at school A.

‘Everyday treats’ thus included a range of different social practices linked to the everyday life of the family, but these practices were less culturally codified compared with the practices linked to ‘family treats’.

#### 3.2.4. Strategies for Behavioural Changes in Relation to ‘Everyday Treats’

The most common approach to reduce sugar-rich foods in lunch packs and afternoon meals was a substitution strategy, whereby sweet snacks were replaced by healthier alternatives.

*“I give it more thought now what I put in his lunch pack. I have swopped some of the spreads. He really likes chocolate spread, but now he rather gets fruit cuts, or fruit sticks instead of Kinder milk slices”*,

mother to boy at school B.

In the home-use intervention tools, it was explained how a range of frequently used snacks like granola bars, biscuits and cordial should be categorized as sugar-rich along with more commonly recognized sugar-rich products like chocolate, ice cream and sweets. This awareness of sugar-rich snacks motivated swops and new habits in many families.

*“Typically, there was a packet of biscuits in the drawer and that was what the kids wanted when they got home from school. They got two-three-four biscuits according to their appetite. Now we realize the amount of sugar in those biscuits and we are happy to have that knowledge”*,

mother to girl at school C.

*“We do know that cordial contains sugar, but then you read it and it is a kind of an eye-opener […] Now cordial is only served on weekends”*,

mother to girl at school D.

The availability of healthy snacks at home was key in these substitution strategies, and the real change was often made in grocery shopping habits.

*“We limit the amount of unhealthy food when we shop for groceries and serve something else. Afternoon snack are bell pepper and fruit instead of fruit sticks, biscuits and the like”*,

mother to boy at school A.

The altered provisioning consisted of two approaches: either one where a stock of healthier ready-to-serve products such as a fruits and berries, nuts, dried fruits or crispbread replaced, e.g., biscuits or granola bars; or an approach where raw vegetables or fresh-cut fruit substituted the sugary snacks. This change was more work-intensive for parents, as it demanded preparation time and planning.

*“Perhaps we have gotten a little better in preparing veggie snacks and between meals and not necessarily use granola bars or ice cream as our go-to solution. Simply getting these carrot and celery sticks cut (laugh) so that you can serve them in time”*,

mother to boy at school C.

Parents who adopted this approach reflected on the fact that the needed preparation time was closely linked to the overall level of time and resources in the family and thus that there was a risk of slipping back into old habits.

*“The afternoon meal is healthier, but that is also linked to my parental energy level; if I am tired the servings drop in quality, but in general, meals have improved”*,

mother to girl at school C.

As a rule, whether it was in relation to substitution or availability strategies, parents did not transform family habits in any radical way; they just changed them slightly in a healthier direction, while the actual practices remained close to existing routines. Hence, new habits were old habits, just adjusted to be more in line with the intervention messages. 

In contrast, changes that demanded more effort, e.g., that parents change their habits, were reported to be harder to implement. Behavioural changes in relation to carbonated soft drinks were the most frequent issue. Parents’ recognition that they were direct or indirect role models for their children motivated them to change habits and reflect on how they could avoid passing on unwanted habits that they have difficulty controlling in their own lives.

*“I have a guilty pleasure: soda and energy drink. That is my weakness […] And energy drink is the worst; that is really something you should not introduce your children to, right? It makes me think: ‘Wow! My kids should definitely not have energy drink’”*,

mother to girl at school A.

In the families that reduced their daily habitual carbonated soft drink consumption, the behavioural change often also consisted of restricting accessibility. Participants explained that easy access to carbonated soft drinks, e.g., stocks in the fridge or bottles on the dinner table, made reductions in intake challenging, and they thus changed the accessibility.

*“My husband is very fond of soda. He is crazy about real Coke [with sugar] and we have decided that it does not work! We need to stop that, because [name of child] started to grow fond of it too. It was right there, as an alternative [to water] on the dinner table. So we have cut out soda and cordial completely during the week”*,

mother to boy at school A.

The fact that cordial, both artificially sweetened and sugar-sweetened versions, was to be restricted in line with carbonated soft drinks came as a surprise to participants. Parents used to serve cordial—artificially sweetened and to some extent sugar-sweetened—in good faith, but the increase in knowledge obtained through the intervention materials spurred a motivation for behavioural change.

*“That is our biggest change: Water on weekdays!”*,

mother to girl at school D.

Restricting cordial might be seen as a substitution strategy in line with the swops made in relation to lunch packs and afternoon snacks, as cordial is substituted by water or milk. Overall, social practices related to ‘everyday treats’ (with carbonated soft drinks as an exception) were perceived as relatively easy to change.

*“It became clear that there were some things that relatively easy could be swopped with other things”*,

father to boy at school C.

Children’s packed lunches and afternoon snacks (foods and drinks) seemed to be relatively low-hanging fruit in parents’ experience of behavioural change, and new habits could thus be adopted smoothly. 

#### 3.2.5. ‘Socialized Treats’: Sugar-Rich Foods and Drinks as a Cultural Norm

The offering of sweet treats and soft drinks during seasonal celebrations like Halloween and Christmas is a cultural tradition in Denmark. At school, it is tradition that a birthday child brings sweets or cake, and as a rule, festive, social occasions are celebrated with sugar-rich products. In Denmark, shared social events and special occasions thus constitute an important social context for serving sweet treats to children.

Parents reported that they generally had difficulty in regulating or restricting the serving of sugar-rich foods and drinks outside the home. In relation, when filling in the dietary record that was part of the data collection for the quantitative evaluation of the intervention (see Bestle et al. 2020 [26]), many realized the amounts of sweets, cake, soft drinks and the like that their children were served by others, e.g., in school, at leisure time activities or when visiting friends.

*“I was shocked how little control I have over what my child eats during the day […] I mean we can have a healthy balance [with a limited amount of sweet foods and drinks] at home. But then it is a week with a lot of activities: Baking cake in the after school club, and birthdays in class where friends bring Kinder surprise eggs”*,

mother to boy at school A.

Parents emphasized social gatherings and seasonal celebrations as particularly challenging. These traditions often involve sweet treats and often in amounts that the parents would not offer at home.

*“Now it is Fastelavn [Danish Carnival tradition in February] and I think it is exaggerated: They have a bun with icing and cream, and cocoa and sweets. Apparently, they need it all. And at birthday celebrations they wallow in sweets again—and cake and the like. In fact, it is more the things that I cannot change that I think should be cut down”*,

mother to girl at school A.

Other interviewees related that seasonal celebrations, especially Halloween, Easter and Christmas, often resulted in large amounts of ‘left-over sweets’: treats and gifts that relatives had brought for parties or social gatherings that subsequently became unwanted stockpiles.

*“I have used quite some time clearing it all out. The girls have several very ‘generous relatives’ [sarcastic voice], my aunt for example, every year she arrange this Easter hunt, and I mean it is an enormous bag with chocolate eggs”*,

mother to girl at school A.

Another, and related, recurrent topic in parental accounts was sweets and treats served in relation to family relations or social activities. Notably, grandparents were mentioned by the interviewees as a challenge.

*“The hardest thing right now is Grandma. It is really…[sigh]. When they are at grandma’s place they are spoiled. Preferably with sodas and sweets”*,

mother to girl at school A.

Sugar-rich foods and drinks served in relation to social relations or events outside the home thus played a major part in the children’s intake of sweet treats.

#### 3.2.6. Strategies for Behavioural Changes in Relation to ‘Socialized Treats’

Despite their disapproval of the frequent and large amounts of socialized treats served to their children, parents in general did not interfere with social occasions or set special rules for their children in an attempt to limit their intake, e.g., at birthday parties.

*“I mean we are not the kind of parents that tell our kids: “You are not allowed to eat the treats offered at school”, or tell our parents that they should not serve cake when the grandchildren are visiting. There are other considerations to take into account—both in relation to our kids but also family and friends. Concerns that overrule the guidelines [from the project]”*,

father to boy at school C.

Many parents actively chose not to engage in arguments with their parents (or parents-in-law), as they considered it overly difficult or feared that such critiques could cause disagreements that served no purpose. Others addressed the issue.

*“I pay more attention to what my parents offer to the girls, and I have asked them to cut down. I have been more outspoken, than before, because I think they exaggerate the treats. Earlier I have been thinking: “We are not there that often, it will be okay,” but this time I raised it and explained that they should not have all kinds of sweets and treats, when we are visiting”*,

mother to girl at school B.

In relation to the school setting, several parents explained that they would have liked to discuss and establish agreements with fellow parents on, e.g., the amount of sugar-rich foods and drinks offered for social class events like birthdays. However, because of the COVID-19 lockdown, parents in many cases did not get to meet one another for the first 7–8 months of their child’s schooling. In Denmark, there is a strong tradition of parents’ involvement in their children’s schooling, and a central principle of Danish education is that pupils attend permanent classes, thus having the same classmates and class teachers for years [38]. Without the COVID-19 restrictions, parents and teachers would have had several pre-school meetings and social events establishing the social environment of the class.

### 3.3. Barriers for Behavioural Changes

In the analysis of the interviews, a range of barriers to reducing the intake of sugar-rich foods and drinks was identified. A number of barriers related specifically to ‘family treats’, ‘everyday treats’ and ‘socialized treats’, while other barriers intersected these social practices. In the following sections, barriers will be described and organized in the following categorization: (1) culturally codified practices and social concerns, (2) parents’ personal preferences, (3) parents having disparate views on health, (4) lack of motivation, (5) lack of skill and knowledge and (6) lack of parental resources.

#### 3.3.1. Culturally Codified Practices and Social Concerns as Barriers to Change

Across the different social practices related to sugar-rich foods and drinks, ‘socialized treats’ emerged as very hard to change. Parents experienced a lack of control of and influence on what their child was offered as a main barrier. To impose restrictions and limitations on ‘socialized treats’ in relation to family, classmates, friends and other social relations was perceived as in conflict with social and cultural norms, and social concerns took precedence over the dietary guidelines.

A comparable attitude was seen in relation to ‘family treats’. The majority of participants reported that family time and sugar habits were associated. To have family quality time often implied some sort of ’comfort food’ high in sugar. This culturally established norm shaped practices and mindsets notably during weekends and holidays. Several families reported that holidays were special, and that things could ‘go a bit off track’. Families deemed it to be expected and acceptable as long as they got back on track when they returned to everyday life.

*“It has been a little less strict, because it is the holidays. I mean, that is when they should feel that you have some extra fun together. The holidays are where you should try to relax and have a good time together”*,

mother to boy at school A.

The main approach in families was thus that sugar-rich foods were acceptable and suitable on family occasions, and if they had made changes to reduce intake, they were generally adjustments (most often of portion sizes) and not restructurings of the concept of, e.g., Friday sweets.

Likewise, it could be socially difficult merely to omit sweet snacks from packed lunches. Some parents reported that the child found it difficult and sometimes embarrassing to explain to classmates why they did not bring the same foods.

*“I mean, why are we so strict (laughs) compared to her classmates and what they have in their lunch packs. We talk about why it is this way, because obviously she finds it extremely annoying […] because if she has to explain in class why she is not allowed, e.g., Kinder milk slice or whatever. Then it is easier for her to say: ‘Well, in our family we think it is like sweets, and we only have sweets on Fridays or at special occasions’”*,

mother to girl at school B.

In the interviews, these concerns were only addressed by parents for whom the importance of healthy food outweighed social norms and expectations concerning lunch packs. It might thus not appear as a barrier, but parents’ explicit efforts to omit these products prove it as such.

Against this rather complex background of divergent social, cultural and dietary concerns, parents tried to balance health objectives and the importance of belonging in social life, e.g., the unity built with classmates, often evolving around shared meals and sweet treats. However, cultural habits and social norms around sugar-rich foods and drinks constituted a major barrier to reducing the intake of sugar-rich foods and drinks in the context of close relatives, family and friends and other social relations. 

#### 3.3.2. When Parents’ Own Habits Inhibit Behavioural Change

As mentioned, reductions in intake of both sugar-sweetened and artificially sweetened carbonated soft drinks proved difficult and constituted a major barrier to behavioural change. Changes demanded that parents change their personal habits, as it seemed hypocritical and inconsistent to them to prohibit children to have carbonated soft drinks but continue such consumption themselves.

*“I drink water all day at work, and when I get home, I just have to have something else, and then it is a bit difficult to say: “No, you cannot have soda, but mom can”, right?”*,

mother to boy at school B.

Parents’ habits were reported to have a large influence on children’s access and consumption patterns, and if they were not motivated to reduce their intake, their children’s intake did not change either. Some families ‘customized’ the guidelines to their own habits. These families approved of the guidelines and the intervention materials as such but made omissions in relation to artificially sweetened beverages.

*“In our family, we think sugar-free cordial for dinner is okay […] We as parents have a different definition [than the project’s] of what counts as sugar and what does not count as sugar. We don’t include sugar-free soft drinks in the ‘sugar account’. We don’t deem it to be sugar”*,

mother to girl at school C.

However, parents’ preferences also constituted a barrier on a more general level and not only in relation to soft drinks. Interviewees reported similar practices in relation to other preferences of their own.

*“But it is also because I have chocolate spread myself, and you know… perhaps I should not, but I like it for breakfast. Then obviously, she wants it too, that is fair enough”*,

mother to girl at school A.

Comparable habits were elicited from the interviews, e.g., being fond of ice cream or biscuits and thus having them stored, which often led to children having them as treats on a frequent basis. The same pattern was identified in relation to lunch packs, as parents’ habits influenced decisions concerning what to prepare or serve for one’s child.

*“I enjoy giving them small treats. Or perhaps not treats but something nice. Sweet biscuits for example, well in fact it often turns out to be some kind of lousy carbohydrates that I have put in […] I think it is because I myself like to have something yummy after my sandwiches”*,

mother to girl at school C.

Parents’ preferences and personal habits, with carbonated soft drinks as a case in point, played a pivotal role in the development of their children’s preferences and constituted a major impediment to reducing the children’s intake of sweet treats.

#### 3.3.3. Parents with Disparate Views and Perceptions of Healthy Habits

Health perceptions and related practices within families differed significantly across the sample, but in some families, they also differed between parents. One interviewee explained how lunch packs differed markedly based on whether she or her partner prepared them. She made an effort to make lunch packs nice and appetizing, while he was more prone to choose easy-to-prepare solutions. Other mothers reported the same pattern.

*“They cannot bring small cinnamon rolls and stuff like that […] Fruit sticks, raisins and the like is okay. And they can also bring granola bars, I know they are not particularly… but he gets them quite often. When I do the lunch pack I skip them, but I know my husband gives them (laugh). We are not totally aligned, when it comes to food”*,

mother to boy at school A.

Disparities in attitudes and practices created family dynamics in which parents were pitted against each other in more or less opposed roles, which became a barrier to reducing the intake of sugar-rich foods and drinks.

*“He is convinced that we are quite well off, I think we could do better (laugh). That said, I do not want to always be the super strict one, but it is always him buying McDonald’s. It is always him that offers an ice cream after dinner. It is never Mom […] my husband is very fond of easy solutions”*,

mother to boy at school A.

*“It is entirely me who is responsible for this hustle and bustle of health in our family, [name of husband] he drinks coke and eats crisps”*,

mother to boy at school B.

In all families where parents did not agree on a mutual family approach to the guidelines and ensuing behavioural changes, the disparities led to compromises and inconsistencies.

#### 3.3.4. Lack of Skill and Knowledge as a Barrier to Reductions 

A barrier to the substitution strategy often employed in relation to ‘everyday treats’ was a perceived lack of healthier alternatives to sweet snacks. Parents expressed that they have difficulty navigating the intricacies of ‘healthy snacks’ offered, e.g., in supermarkets. Aware that the industry often labels highly processed products specially designed for children as ‘healthy snacks’, parents were left confused as to whether the products were truly healthy.

*“I can on a daily basis be confused, what is this? It says that it is apple, but I mean it is not an apple. It is shaped as an astronaut, and honestly, I have no clue what it is”*,

father to girl at school C.

As the intervention materials handed out to the families present only a certain range of examples of serving sizes for the most popular sweet treats (e.g., chocolate, sweets, biscuits, ice cream, etc.) some parents expressed that they felt that they were left ill-equipped for making informed, healthy choices unless they avoided processed, sweet snacks altogether. However, to omit processed, sweet snacks potentially demanded time-consuming preparation of other snacks (see Section 3.2.4 for examples) or was hampered by the child’s expectations linked to social norms and concerns (see Section 3.3.1).

#### 3.3.5. Lack of Motivation

Another barrier identified among a few families was a lack of motivation to make changes due to having ‘skinny’ children. Parents expressed wonder and hesitation when faced with the school health nurse’s advice on reducing intake of sugar-rich foods and drinks.

*“I was surprised that there was an issue [with sweet foods and drinks], because he is so thin […] he is clearly both small and skinny, so it was hard to believe that he had too much sweets. I mean, I did understand what she was saying, but to go so far as to say that he had too much. I mean, you cannot tell from looking at him”*,

mother to boy at school B.

These interviewees were all mothers who reported having struggled with their children being skinny and accordingly had made an effort to make their children eat a calorie-dense diet. In this context they considered the school health nurse’s instructions irrelevant for them and rejected most of the advice.

*“When I look at him—I mean his is skinny-skinny. There is not one gram of fat on his body. Therefore, I have not been overly engaged. You know, there are some foods I have replaced, but not a lot”*,

mother to boy at school A.

Other families with children experiencing lack of appetite received the advice positively and conceived of it as placing increased importance on eating a healthy and nutrient-dense diet because their child ate small quantities. Thus, that children were skinny did not arise as a certain barrier to motivation and ensuing changes in behaviour, but only emerged in some families. 

#### 3.3.6. Lack of Parental Resources and Prioritization 

Limited time and resources determined the engagement and efforts to change habits in many families. Interviewees related how behavioural change required resources and investment in establishing new, healthier habits, thus making changes susceptible to failure if time or resources declined.

*“We are not there yet. We still need to focus and cannot wriggle out of the risk for backslide. Especially if our mental energy is drained”*,

father to girl at school C.

Several parents raised comparable concerns, and the COVID-19 situation further conditioned sustained changes. Many families experienced that the lockdowns and extended period of home-schooling turned their everyday life upside-down.

*“The main aim here is to change some routines in our everyday life, but we did not really have ‘an everyday life’, at least not as it used to be [laughter]. That might have made it a bit harder”*,

mother to boy at school C.

In general, families reported that when routines dissolved, normal, healthy patterns regressed, e.g., lunch and snacking choices. In this case, it was due to the COVID-19 pandemic, which was an in all regards an exceptional situation, but the logic and experienced vulnerability to lack of time and resources was a general worry to the majority of families.

In a small subset of less advantaged or ‘socially challenged’ families, another more resource-dependent pattern emerged. In all families, parents struggled to preserve their limited resources and tried to prioritize parental core values and translate them into parental practices, trying to balance resources and concerns. However, for families in the aforementioned sub-set, social challenges or lack of time and resources were markedly more severe than in the rest of the sample.

*“I also think it depends what kind of family you have. If we only had one or two children, it would not have been so challenging for us, because then we would have had the resources and would have been able to stick to it. But now… when the daily hustle and bustle takes over and you get more and more busy, you know, which battles do we choose”*,

father to boy at school D.

Parents were emotionally and practically engaged in mitigating challenges that were of greater importance to them than dietary concerns. Challenges varied but centred on children who for one reason or another were not thriving, such as the son of this interviewee who had been struggling with school avoidance.

*“I am too exhausted for such things. I need to walk him through the mandatory stuff [curriculum] because he is at home. And he is not having the same input and stimulation that he used to have when he was attending school and the after school club. It is sheer hard work to home-school him”*,

mother to boy at school A.

Parents’ choices are grounded in their everyday realities, where they consider challenges and try to resolve stressful situations and provide care and well-being to their children where they believe it is most needed. Several parents in this group had had higher expectations for the health outcomes of their participation in the intervention, but at the evaluation interview, they recognize that nothing had really changed.

*“I think perhaps that I could have put in more effort. But then… at the same time I think that the time simply was not there. There has just been too much going on”*,

mother to boy at school A.

These parents did not disapprove of the guidelines as such, but their main parental concern was to provide comfort and support for their children, and they were, therefore, reluctant to enforce reductions in sugar-rich foods and drinks.

### 3.4. Approaches to Behavioural Change and New Habits

Across the social practices related to sweet treats and the identified barriers to changes, two different overall interpretations of the guidelines on sugar-rich foods and drinks emerged in the interviews. These two approaches were defined by how parents construed the guidelines in relation to how their parental core values were identified, and they will be described in the following section, along with how these perceptions impacted parental attitudes toward behavioural change and new habits.

One approach that accounted for a larger part of the interview sample was a strategy where parents made an effort to pursue the guidelines ‘to the letter’, in the sense that they tried to reduce their child’s intake to the maximum-limit of four weekly servings. The serving size board and the inspiration booklet were designed to make families aware of their children’s intake and facilitate reductions enabled by the logic of servings that could be counted. Although it could be difficult to reduce intake to four servings, it was still their objective.

*“Sometimes she exceeds the four servings over the course of a weekend. She does”*,

mother to girl at school B.

In order to not exceed the prescribed limit of four servings, it was common that parents improvised and diverged from the determined serving sizes or decided that certain foods or drinks ‘did not count’, e.g., cordial or sweet treats served when spending time with grandparents. The most common way to remain in line with the maximum of four servings was to ‘understate’ the number of actual servings.

*“My aim has not been to stick strictly to four servings. It has been close to four servings and should one or two more be added without our influence, then that is okay”*,

mother to girl at school C.

As such, the guidelines and the materials designed to support the quantification of intake as ‘servings’ functioned as intended, though parents often made small adjustments in order to make the behavioural changes feasible in their everyday family life settings. 

In the other approach, parents perceived the guidelines more as a rule of thumb for healthy eating and did not conform to the maximum number of servings but employed the logic of limiting the child’s intake on a more general level. Several interviewees stated that counting sweet servings on a weekly basis seemed futile to them, as weekly intakes differed greatly. Hence, they preferred to employ a broader perspective where they kept track of intakes and tried to balance them on, e.g., a monthly basis. Others deemed the counting and ‘accountant logic’ of four weekly servings to be too rigid. Thus, they kept an eye on the amounts, but without counting servings.

*“Then you count the different servings and such. That would be too formalized and rigid in my opinion”*,

mother to boy at school A.

Interviewees expressed that the guidelines were relevant and had provided them with a higher awareness and vigilance in relation to their children’s sugar habits, but it was either unfeasible or irrelevant for them to count the actual number of sweet servings.

*“It has been a wake-up call, and we have greater awareness, but the guidelines are so strict that we did not aim to adopt them (laughter)”*,

father to boy at school C.

The guiding principle of the new guidelines is a clear definition of the food group of sweets, including desserts, biscuits, granola bars, sweet spreads and similar sugar-rich products. The guidelines induced awareness and vigilance regarding these categorizations and prompted concerns as to which food groups should be considered as ‘sweet’ other than obvious items such as sweets and chocolate.

*“We have used it in assessing quantities and to estimate “what is actually sweet?”. As in, what should be counted in. After all, sweet treats are not just sweets. It is also dried fruits and cakes and—yes, we have used it in that way, rather than: “How many servings does she get a week?””*,

Mother to girl at school B

In the families that interpreted the guidelines in a more general sense, the intervention materials had sparked family discussions on diet and well-being. These families reported that the materials and tools had served as an inspiration and framework of reference. They appreciated the all-family approach and reported improved health literacy and higher awareness of healthy habits and family routines. Thus, the relatively narrow focus on sugar-rich foods and drinks seemed to have had a ripple effects across other health domains.

## 4. Discussion

The ‘Are you too sweet?’ intervention aimed to motivate and facilitate a reduction in the intake of sugar-rich foods and drinks among pre-school children. Based on the thematic content analysis, three key domains of social practices among participants were identified: (1) ‘family treats’, (2) ‘everyday treats’ and (3) ‘socialized treats’. Parental strategies employed to reduce children’s intake included substitutions, portion-size reductions and limiting home availability. Overall, the interviewed families reported perceived changes in a diverse range of practices, notably concerning ‘everyday treats’ and to a lesser extent ‘family treats’, while the fewest changes were seen with regard to ‘socialized treats’. While some families made radical changes, most settled for mere adjustments. 

### 4.1. Easily Adoptable Reduction Strategies Requiring Less Effort Were the Most Likely to Be Implemented

Families most frequently made changes when the new practices were easily adoptable and were mere modifications of existing practices, e.g., swopping sweet treats for healthier alternatives in packed lunches and afternoon meals, or reducing portion sizes of ‘family treats’ while leaving the overall concept unchanged. Biscuits, granola bars and other sugar-rich snacks were relatively smoothly substituted by crisp bread, fruit or vegetables in most families, as doing so primarily required parents to shop differently and thus demanded manageable efforts. For some parents, it was still experienced as challenging to find healthier alternatives, illustrating a demand for increased availability of healthy snack foods, e.g., in retail stores or school settings. Changes that demanded more substantial efforts, for instance, that parents change their daily habits by, e.g., reducing their personal intake of carbonated soft drinks, were less common. 

Other studies investigating successful behavioural changes confirm that making healthy adjustments to existing habits and routines is effective, and participants consider changes which are convenient to implement to be favourable [39,40]. In a preceding evaluation among the participating families of the ‘Are you too sweet?’ intervention components, it was likewise found that tools that fit into existing, practical everyday habits were more commonly employed and frequently used [19]. 

More codified cultural events encompassed in ‘family treats’ and their related practices, like Friday sweets, demanded other strategies, as the events were socially conceptualized to involve sugar-rich foods and drinks. Friday sweets play an important role in Danish family culture and are integral to ‘family treats’ in many families. Again, behavioural changes more commonly involved adjustments of, e.g., portion sizes, than substitution of sweets with healthier choices or other activities. Families who had chosen to have Friday sweets took pleasure in the tradition and were not motivated to change it drastically. For ‘family treats’ more broadly, the concept of Danish ‘hygge’ carried an important cultural meaning and indicated a cosy time with the family, notably during weekends and holidays. On such occasions, sugar-rich foods, snacks and drinks were an integral part of the ‘cultural script’ [41] and thus not easily changed or substituted by other food items or activities, as doing so would corrupt the ‘cosiness’ (‘hygge’). Studies of both ‘hygge’ and how it is created [42] and of consumption patterns in Danish families [15] describe comparable social meanings and practices around weekends and family time, where ‘hygge’ with sweet treats appears as a marker of ‘real’ family togetherness, linking particular social practices with specific foods [18,42].

### 4.2. Cultural Events and Social Concerns

Cultural norms around celebrations, festive events, and social gatherings where ‘socialized treats’ were served—sometimes in huge quantities—were reported as challenging by participants. Social concerns were reported to be an impediment to reducing intake of sugar-rich foods and drinks. Parents explained that they regularly needed to balance the maximum limits against considerations of social norms. Social concerns most often took precedence over maximum limits, and health concerns were side-lined in favour of following the social norms of commensality and food ways in social relations. Similar findings have been reported in the literature on dietary behavioural changes and reduction of intakes of sugar-rich foods and drinks, notably in relation to family-based childhood obesity prevention (e.g., [43,44,45]). Several parents report the same ambivalence when their child spent time with relatives, e.g., grandparents, and disregarded health concerns in order to avoid perceived counterproductive conflicts. Similar dilemmas between health and social concerns are common in the literature [46,47]. The processes and practices of changing habits around sugar-rich foods and drinks are embedded in social life and thus linked to a range of concerns that parents seek to balance—health not necessarily being the most important.

### 4.3. Reduction Strategies and Barriers to Behavioural Change

The results thus indicate that strategies of substitution, portion size reduction, availability and accessibility of heathy snacks and interrelated new shopping routines can prove effective if the changes are easy to embed in current family practices and only demand small adjustments. However, families experience different barriers to achieving reductions in the intake of sugar-rich foods and drinks that cut across the areas of ‘family treats’, ‘everyday treats’ and ‘socialized treats’. These included (1) culturally codified practices and social concerns, (2) parents’ personal preferences, (3) parents having disparate views on health, (4) lack of motivation, (5) lack of skill and knowledge and (6) lack of parental resources, i.e., trying to balance resources and concerns. 

That parents’ own preferences were a barrier for their children’s behavioural change became particularly clear in the case of reductions in intake of both sugar-sweetened and artificially sweetened carbonated beverages, as parents’ habits had a huge influence on children’s access to such drinks, and if parents were not motivated to reduce their own intake, children’s intake did not change either. A comparable degree of impediment to change was found in relation to ‘socialized treats’ that due to the cultural codification are closely linked to social norms that overrule dietary concerns. The ‘Are you too sweet?’ intervention was designed as a family-centred, home-based program, and accordingly, intervention components did not provide participants with tools or strategies aiming to reduce intake of sugar-rich foods and drinks in relation to social relations or events outside of the home. Yet, as social norms largely drive individual behaviour [48], the results call for a supplementary intervention modus, delivery mode and messages targeting macro-level and structural changes of social norms around sweet treats, celebrations and sociality. 

Two overall parental approaches to the recommended maximum intake of sweet treats were identified among interviewees, but despite the differences in the two groups, parents approved of the guidelines and implemented changes to varying degrees. 

In the paradigm of ‘New Public Health’ [49,50], the emphasis shifts from preventing disease to promoting health [51]. The main objective is thus to facilitate health promotion and preventive measures that support societal structures in which people can maintain and improve their health and well-being. The ‘Are you too sweet?’ intervention was designed in line with such an approach, as the aim was to improve diet quality over a broad spectrum of children. A large proportion of interventions directed at high intakes of sugar-rich products, e.g., in the framework of obesity-prevention [52,53], frequently target selected sub-groups (vulnerable families, ethnic minorities, children with obesity, etc.), while the ‘Are you too sweet?’ intervention aimed at facilitating behavioural changes through a population approach [54,55]. The analysis of the interviews suggested that among most families, an increase in knowledge and awareness regarding sugar-rich foods and drinks (e.g., the recommended maximum number of servings, and that biscuits, cordial and granola bars all are sugar-rich products) facilitated the development of healthier family habits and consumption of sweet treats in moderation—regardless of, e.g., weight status. Parents described how they appreciated being made aware of the possible health consequences of high intakes of sugar-rich products and welcomed the strategies for behavioural change. However, a small sub-sample of families did not succeed in reducing intake of ‘family treats’, ‘everyday treats’ or ‘socialized treats’, either due to lack of motivation or resources. To meet the needs of these families, other strategies and intensified attention and care may be needed. For example, Elinder and colleagues [56] argue that in order to reduce social inequalities in health and support vulnerable parents, interventions should be tailored to the special needs of families.

### 4.4. Strengths and Limitations

It is a major strength of the present study that due to the predetermined large sample size (n = 24), a broad representation of different family types among the interviewees was obtained. Different parental practices and strategies in the interview sample enabled a rich and nuanced analysis. Notably, that more socially challenged and vulnerable families were also a part of the sample allowed for insights into this group, their resources and their prioritizations. Yet, fathers are still under-represented among the interviewees despite sustained efforts to recruit them, and in-person interviews would have been favoured over the online versions imposed by the COVID-19 pandemic.

However, the fact that the intervention had a follow-up period of 3.5 months and that long-term sustainability of the families’ behavioural changes is thus unknown is a limitation. Another limitation is the potentially limited applicability of the findings in other settings due to the context-specific behaviours linked to particular Danish cultural traditions and norms such as ‘hygge’ and Friday sweets, though similar practices and family values might be found elsewhere [57]. Additionally, lockdowns and the extraordinary circumstances resulting from the COVID-19 pandemic might bias results, as many family routines were partly disrupted during this period. 

### 4.5. Considerations on Research Needs and Future Initiatives

Considerations for future interventions and initiatives include strategies for coping with the barriers identified in this study. Cultural and social norms concerning sugar-rich foods and social events and activities (e.g., portion sizes or the link between certain social occasions and particular foods) are deeply anchored in cultural scripts and therefore hard to change. However, social norms and dietary practices inevitably change over time, and strategies and policies that hold the potential to influence these changes in a healthier direction are important [58,59]. Future research could further focus on developing strategies that expand the adoption of the guidelines on maximum limits among different groups of families and in other countries by tailoring components and main messages in order to make changes feasible and relevant and thereby continue the impact on parents’ behavioural changes. Furthermore, the results indicate the need for intervention strategies addressing barriers that might require structural changes in social norms and values. Such changes in general perceptions and attitudes could in turn promote the development of, e.g., a broader range of healthy snacks and alternatives to sweet treats (and vice versa by offering healthier snack foods promoting a change of norms) and thereby create a virtuous circle. 

## 5. Conclusions

The analysis in this qualitative evaluation showed that reductions in children’ intake of sugar-rich foods and drinks were linked to different domains of social practices. Reductions related to the family’s everyday life (‘everyday treats’) were experienced as relatively smooth adjustments demanding little effort by parents. In contrast, reductions in ‘family treats’ and ‘socialized treats’, proved more difficult, though families reported that strategies of downsizing and portion control had been implemented with success. Yet, ‘socialized treats’ served on special and social occasions carried important normative meaning, and parents found it difficult to apply positive behavioural changes. 

The results indicate that it is important to tailor intervention messages and tools to be in line with social practices around sweet treats. In this study, it proved effective to provide parents with different reduction strategies so that families could customize behavioural changes that fit with their existing family attitudes and practices.

## Figures and Tables

**Table 1:** Summary of the characteristic of the interviewees in the evaluation interviews.

	Population Interviewees, n = 24
Sex of participating child; n (%)	
Girls	14 (58%)
Boys	10 (42%)
Interviewees; n (%)	
Mother	18 (75%)
Father	1 (4%)
Both parents	5 (21%)
Highest parental education; n (%)	
Basic school (<12 y)	3 (13%)
Upper secondary school (12 y)	0 (0%)
Vocational education (13 y, practical)	7 (29%)
Short higher (13–14 y)	4 (17%)
Medium higher (15–16 y)	5 (21%)
Long higher (≥17 y)	5 (21%)

**Table 2:** Key themes and subthemes identified in interviews.

Themes	Subthemes
Sweet treats in social relations	Sweet treats from grandparents
	Sweet treats from close relatives
	Sweet treats served at social occasions
Behavioural strategies for reductions	Substitution strategies
	Strategies on portion size reductions
	Strategies reducing serving frequency
	Strategies reducing availability and accessibility
Barriers to reduction strategies	Culturally codified practices, social concerns
	Parents’ personal preferences
	Parents having disparate views on health
	Lack of motivation
	Lack of skill and knowledge
	Lack of parental resources
Reported behavioural changes	Sugar-rich drinks
	Sweet afternoon snacks
	Lunch packs
	Family weekend and holiday habits
	Other practices

## Data Availability

Not applicable.

## Supplementary Materials

The following supporting information can be downloaded at: https://www.mdpi.com/article/10.3390/ijerph191811647/s1, File S1: Topic guide for qualitative interview.
