# Supplementary material for: A Qualitative Evaluation of Social Aspects of Sugar-Rich Food and Drink Intake and Parental Strategies for Reductions
Source: Int J Environ Res Public Health. 2022 Sep 15;19(18):11647. doi: 10.3390/ijerph191811647 (PMC9517337; doi:10.3390/ijerph191811647)
Supplement: Supplementary file 1 [file ijerph-19-11647-s001.zip › ijerph-1841129-supplementary.pdf]

International Journal of
Environmental Research and Public Health

MDPI

File S1: Topic guide for qualitative interview

The topic guide for qualitative evaluation interview with families was developed to assess the usefulness and acceptability of the intervention components and mode of delivery, and the families' actual behaviour changes in relation to sugar-rich foods and drinks, and the experienced facilitators and barriers for these changes. Additionally, parents' perception of the guidelines on the maximum number of weekly servings and their motivation for and potential experiences with implementing them was assessed.

In this study, the aim was to evaluate acceptability, usefulness and motivational potential of the intervention components, and hence not all topics in the guide have been included in the analysis, but only those relevant for this analysis.

Theme	Content	Focal points	Time frame
Introduction	1. Introduce the aim of the interview<br>2. Inform about the structure and themes of the interview<br>3. Inform about anonymity (no individual identities will be disclosed in publications)<br>4. The audio recording is for internal use only (data protection)<br>6. Oral consent to participate<br>5. Do you have any questions?	Present the aim of the interview.<br>Introduce the logic of qualitative interviews (that interviewee's subjective experiences and perceptions are central, and that their point of view is necessarily right)<br><br>Inform on confidentiality, withdrawal and data protection<br><br>Obtain consent of participation	4 minutes
Overall evaluation	How was it to be part of the 'Are you too sweet?' intervention?	Participant's top-of-mind evaluation and thoughts?	3 minutes
Behaviour change and motivation	Have you changed any habits or practices?<br><br>What did the habits and intake of sugar-rich foods and drinks look like in your family before you enrolled?<br>**Prompts:** Friday night sweets, lunch packs, soft drinks and other sweet drinks, grandparents and afternoon snacks<br><br>**Additional prompts:**<br>1. Experiences with the 7 day dietary registration<br>2. Parental agreement on e.g. rules, habits and/or changes<br>3. Sustainability of new habits (Friday night sweets, lunch packs, soft drinks, portion sizes	Successful behaviour change in family habits and routines<br><br>Experienced barriers, facilitators and motivations in relation to behaviour change.	10 minutes

	and/or frequency of servings) – promoters and barriers		
Knowledge on the guidelines for sugar-rich foods and drinks	Did you acquire knowledge through the project that you did not possess beforehand?<br>**Prompts**: weekly number of maximum servings, portion sizes, definitions of sugar-rich foods and drinks, other?<br><br>What are your thought on the logic of a maximum number of servings?<br><br>Did you aim to comply with the guidelines?<br><br>Did you customize the guidelines? E.g., tailor the number of maximum weekly servings? Other?<br><br>What are the challenges of complying with the guidelines, if any?	The applicability and relevance of the guidelines<br><br>How do parents perceive of the recommended maximum number of weekly servings?<br><br>Experienced barriers, facilitators and motivations in relation to reducing the intake of sugar-rich foods and drinks to the recommended maximum number of weekly servings	8 minutes
Consultation with the school health nurse	What are your thought on the consultation with the school health nurse?<br>**Prompt:** Sugar-rich food screener output<br><br>Which family members participated (child, mother, farther, both) in the consultation?	Perception of the consultation with the school health nurse<br><br>Mode of delivery for main message and intervention components, including the sugar-rich food screener output	5 minutes
Intervention components and tools	If we look at the things in the home-use box with materials, what did you actually use?<br>**Prompts:**<br>1. The serving size board?<br>2. The inspiration booklet?<br>3. The read-aloud children's book? Did [child's name] enjoy it? Did he/she capture the educational health message on dental health?<br>4. The card game (The Monster Game)? Did [child's name] enjoy it? Did he/she capture the educational health message?<br>Did you download the app? Have you/[child's name] used it? Which features have you/[child's name] used?	Practical use of the intervention components, and potential tailoring or innovations<br><br>Pedagogical usefulness of the intervention components<br><br>Practical or technical impediments<br><br>Disapproval or critique of the intervention components	10 minutes

	Did you subscribe to the Facebook group?<br>Did you get notifications on the posts posted? Have you read/seen the posts? What are your thoughts on the content?<br>Did the posts serve as a reminder of your participation in the project?		
The COVID-19 pandemic, lockdowns and restrictions	How did the COVID-19 pandemic and lockdown affect your family and everyday life?<br><br>Do you think you would have been more or less involved in the project if lockdown had not happened?<br>Prompt: E.g., used the things in the box with home-use materials more or less?	To what extent and how has the COVID-19 pandemic and lockdown been a barrier or a facilitator concerning behavior change?	8 minutes
Social support	Did you discuss the project with family or friends?<br>**Prompts**: Other parents from [child's name] class?<br>Close family members, e.g., aunts, uncles or grandparents?	Family networks, core values and norms	4 minutes
Outro	Do you have anything to add? Things that we did not discuss that are relevant to the evaluation?<br><br>Do you have any questions?		3 minutes
